# Supplementary figures and images for: Bisphosphonate drugs have actions in the lung and inhibit the mevalonate pathway in alveolar macrophages
Source: eLife. 2021 Dec 30;10:e72430. doi: 10.7554/eLife.72430 (PMC8718110; doi:10.7554/eLife.72430)

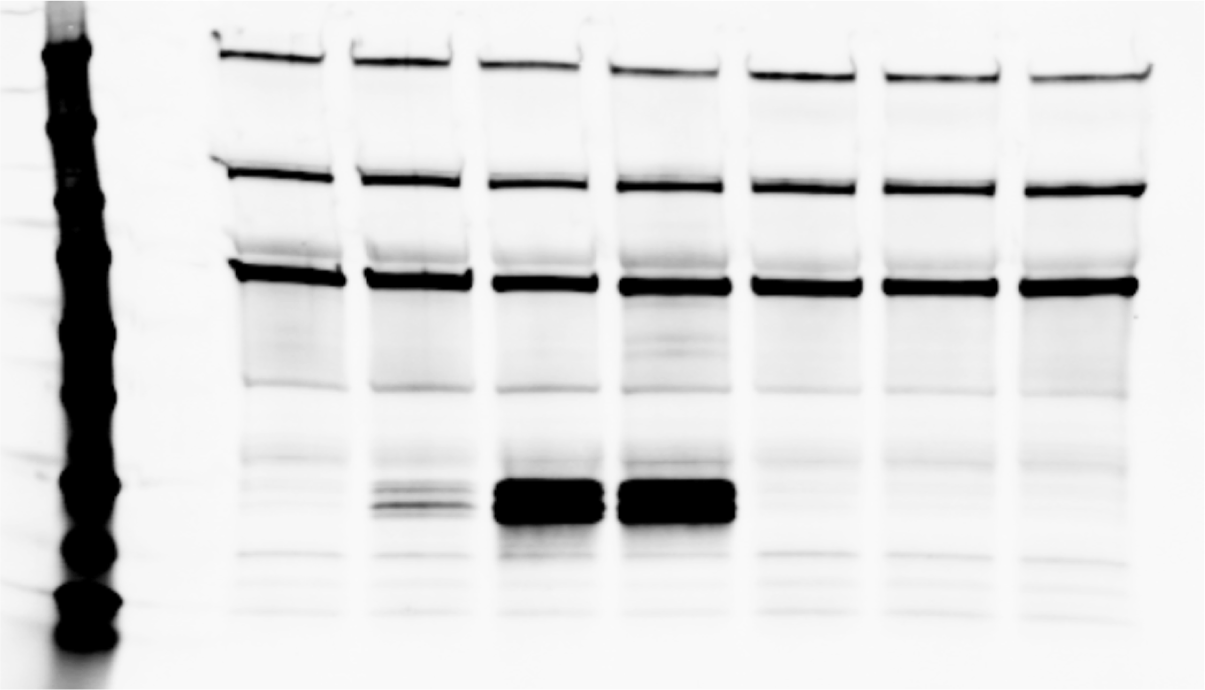

Supplement: Figure 1—source data 1. [file elife-72430-fig1-data1.zip › Figure 1a_source data 1.tif]

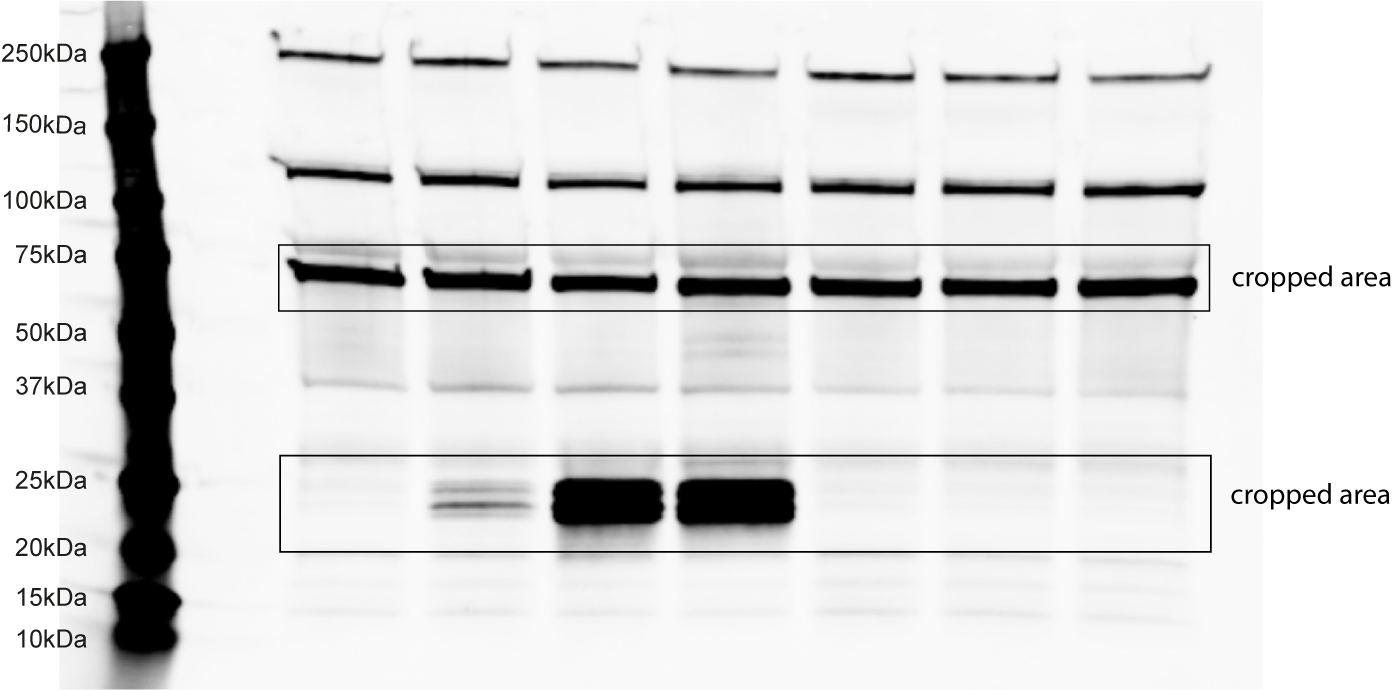

Supplement: Figure 1—source data 2. [file elife-72430-fig1-data2.zip › Figure 1a_source data 2.tif]

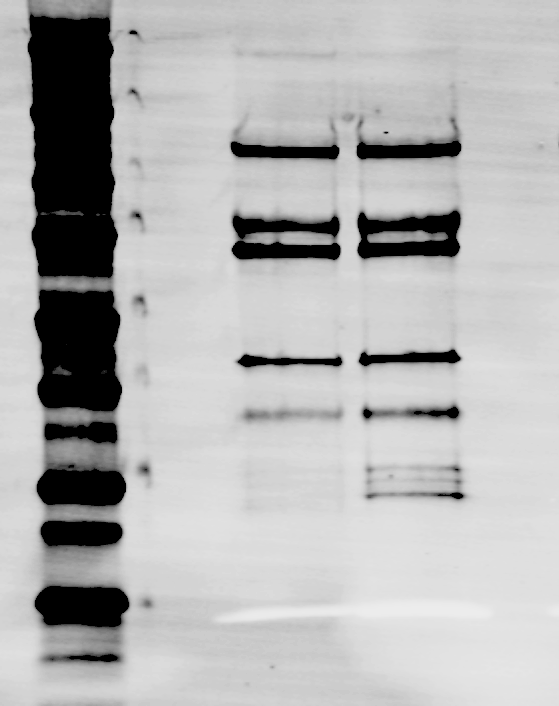

Supplement: Figure 2—source data 1. [file elife-72430-fig2-data1.zip › Figure 2c BAL_source data 1.tif]

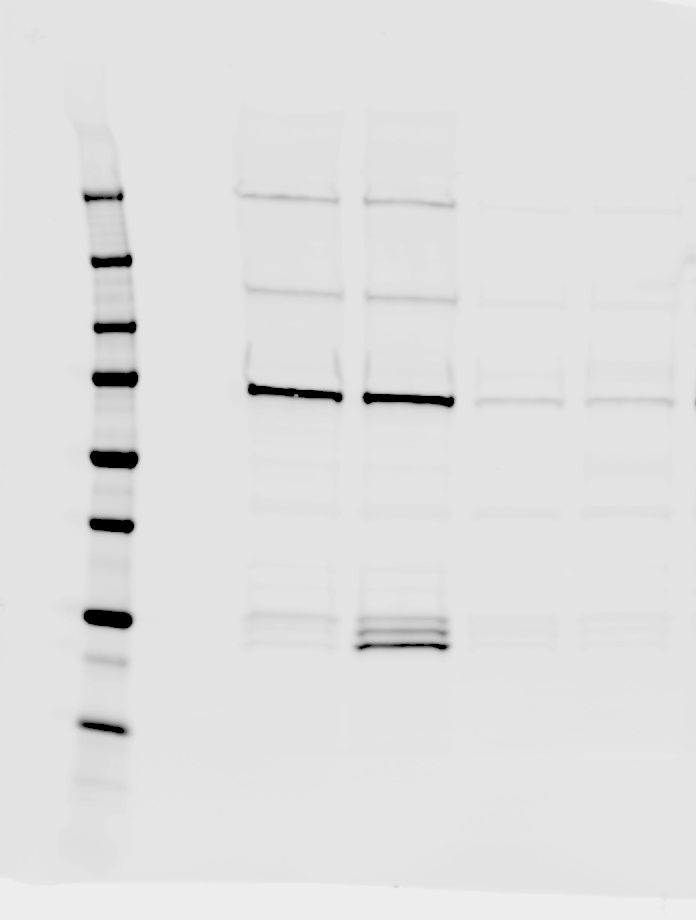

Supplement: Figure 2—source data 1. [file elife-72430-fig2-data1.zip › Figure 2c PL_source data 1.tif]

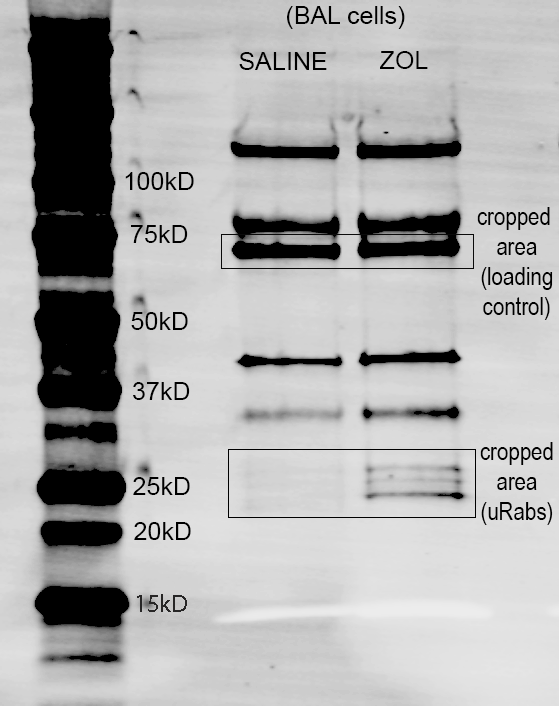

Supplement: Figure 2—source data 2. [file elife-72430-fig2-data2.zip › Figure 2c BAL_source data 2.tif]

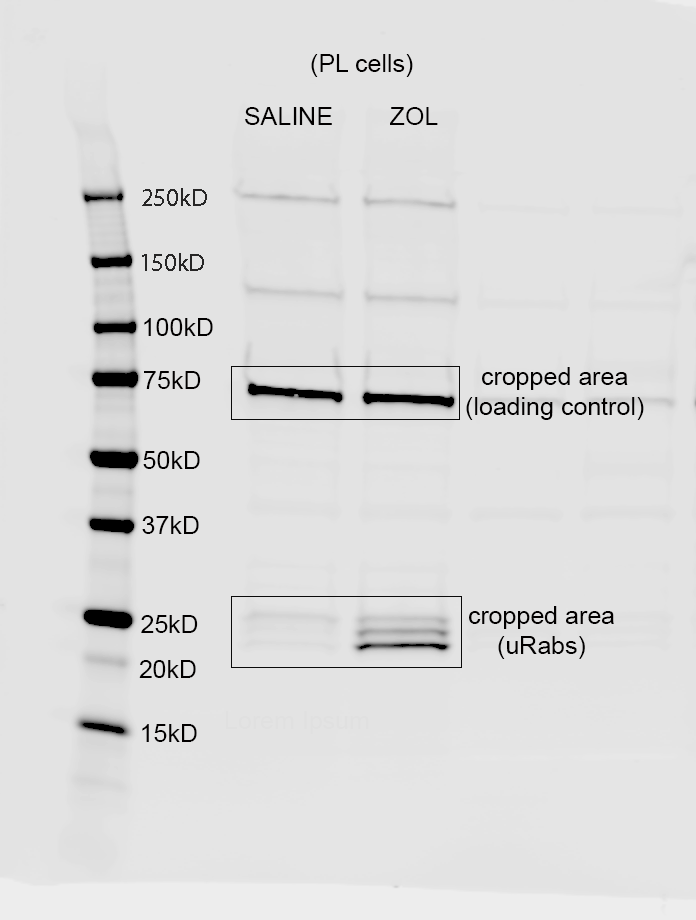

Supplement: Figure 2—source data 2. [file elife-72430-fig2-data2.zip › Figure 2c PL_source data 2.tif]

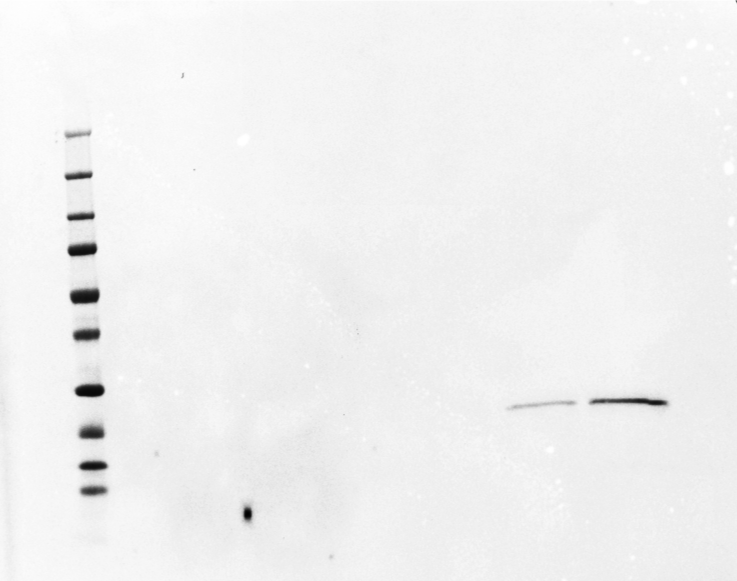

Supplement: Figure 3—source data 1. [file elife-72430-fig3-data1.zip › Figure 3d_source data 1.tif]

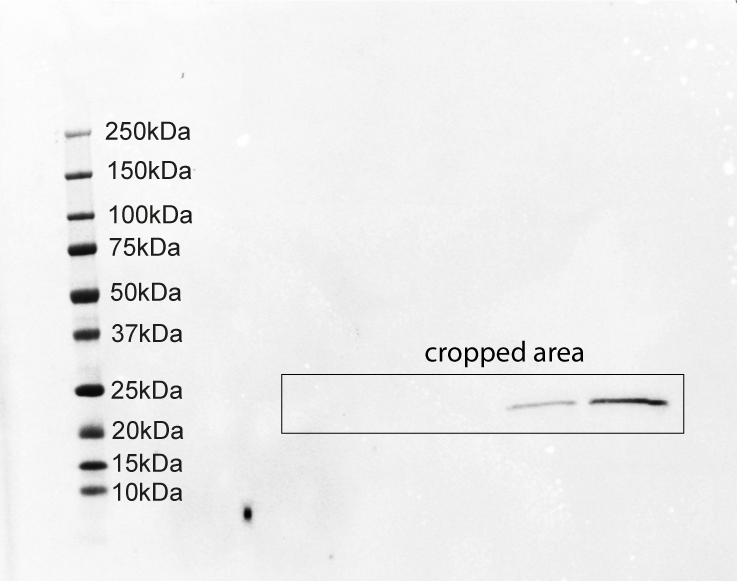

Supplement: Figure 3—source data 1. [file elife-72430-fig3-data1.zip › Figure 3d_source data 2.tif]
